# Supplementary material for: Real-World Effectiveness in Hypertension and Hyperlipidemia Collaborative Management between Pharmacies and Primary Care in Portugal: A Multicenter Pragmatic Controlled Trial (USFarmácia®)
Source: Int J Environ Res Public Health. 2023 Aug 1;20(15):6496. doi: 10.3390/ijerph20156496 (PMC10418740; doi:10.3390/ijerph20156496)
Supplement: Supplementary file 1 [file ijerph-20-06496-s001.zip › ijerph-2232987-supplementary - for XML/SUPPLEMENTARY FILE 1.pdf]

SUPPLEMENTARY FILE 1. Criteria for Intervention and Control Primary Care Units and Pharmacies

Intervention Primary Care Units and Pharmacies

Intervention primary care unit (PCU) selection was based on convenience criteria of expression of interest from the PCU São Julião da Figueira, confirmed by the written authorization statement of the Executive Director of the health center group (‘Agrupamento dos Centros de Saúde’, or ACeS) Baixo Mondego on 18 January 2016.

Intervention pharmacies were selected based on the following criteria:

- Located in the municipality of the Intervention PCU (Figueira da Foz).
- With pharmacy dispensing claims software Sifarma® 2000 2.9.4.3 (Glintt, Sintra, Portugal) and data network Farmalink®.
- Within <1,400 meters of intervention PCU and/or proportion of prescriptions from intervention PCU (on total pharmacy prescriptions volume) ≥15% and proportion of prescriptions from intervention PCU > proportion of prescriptions from other PCUs.
- Active in the National Association of Pharmacies (ANF) Pharmacy Customer Loyalty Program Saúda® *(to enable reimbursement of interventions from this payer)*.

Intervention pharmacies were eligible to take part in the trial if, in addition, they signed a written agreement thereby accepting to:

- Appoint a Trial Site Contact Pharmacist.
- Enroll staff pharmacists in accredited training sessions, workshops, and interprofessional Quality Circles.
- Recruit patients as per the study leaflet.
- Provide patient informed consent as per the study leaflet.
- Provide the intervention as per invitation and study leaflet.
- Provide and record interventions in dispensing claims software Sifarma® as per ICPs.
- Follow confidentiality, privacy regulations, and ethics.

Two sentences for the patient to consent to the exchange of relevant health data from pharmacists to physicians/nurses and from physicians/nurses to pharmacists were added to the Consent Form of the NHS Patient EHR RSE® 2018 (SPMS EPE, Lisboa, Portugal) under this pilot project for the first time in Portugal:

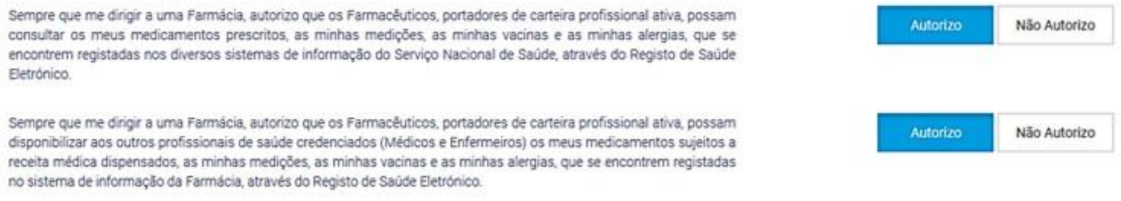

S1FiGURE 1 | Sentences added to the Consent Form of the NHS Patient Electronic Health Record (EHR) RSE® enabling patients to consent to health data exchange with community pharmacists.

Control Primary Care Units and Pharmacies

Control PCU selection involved matching to minimize imbalance: 1) control municipalities to intervention municipality; 2) control PCUs to intervention PCU, followed by selecting control pharmacies, according to the following steps and methods:

1. We used the statistical software R (2018) version 3.5.1. (The R Foundation for Statistical Computing, Vienna, Austria) to estimate three-dimensional normalized Euclidean distance and rank potential best-match control municipalities on:

- Municipality Power Purchasing 2015 Index (‘Índice de Poder de Compra Concelhio’, or IPCC) provided by the National Institute of Statistics (INE), a proxy for economic deprivation.
- Municipality Illiteracy 2011 Rate (‘Taxa de analfabetismo do concelho’) provided by Pordata, census data.
- Municipality Aging 2016 Index (‘Índice de Envelhecimento no Concelho) provided by Pordata.

The graphic representation used R graphic plot.

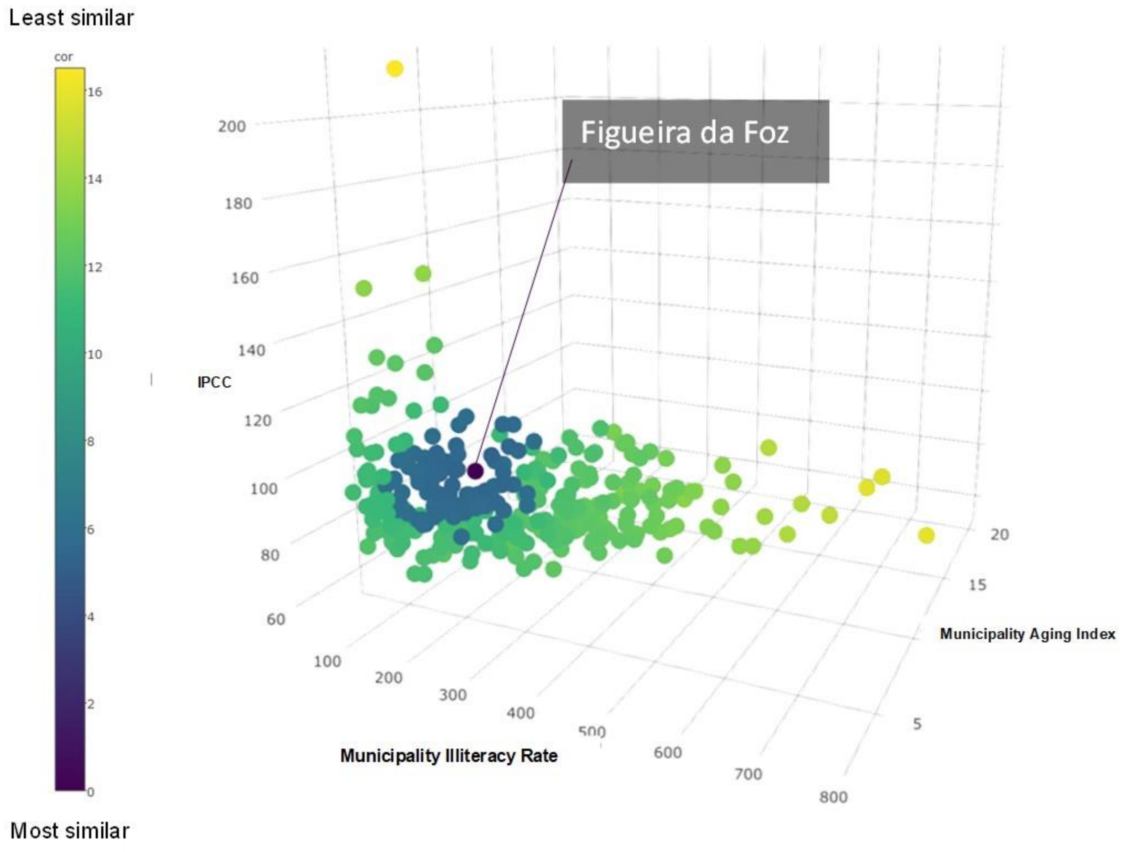

S1FIGURE 2 | Normalized Euclidean distance of each potential control municipality vs intervention municipality according to IPCC, Municipality Illiteracy Rate, and Municipality Aging Index.

2. We selected Predominantly Urban Areas (‘Área Predominantemente Urbana’, or APU), as per the INE classification, in the top 30 municipalities.

3. We mapped potential control PCUs located in those APU areas of 30 municipalities.

4. We ranked potential control PCUs according to match/similarity to intervention PCU on:

- Contractual type “A” PCU (PCUs with institutional incentives if targets are met).
- Electronic Medical Record software SClínico® 2018 (SPMS EPE, Lisboa, Portugal) (USF/ACeS Manual or USF website).
- PCU registered patients' age pyramid in BI-CSP [70].
- Number of PCU-registered patient/physician ratio in BI-CSP [70].
- Proportion of PCU ACeS-registered patients K86 and K87 diagnosis (hypertension), as per the International Classification of Primary Care (ICPC-2) in Perfis Locais de Saúde Região Centro [71].
- Proportion of PCU ACeS-registered patients T93 diagnosis (lipid metabolism disorder) as per ICPC-2 in Perfis Locais de Saúde Região Centro [71].
- Proportion of PCU ACeS-registered patients T82 diagnosis (obesity), as per ICPC-2 in Perfis Locais de Saúde Região Centro [71].
- PCU number of weighted units *(*as a proxy of the PCU burden*)*.

5. We selected the *best match* control PCUs.

6. We, then, selected control pharmacies using the same criteria as intervention pharmacies, except for the Pharmacy Customer Loyalty Program Saúda®. Since these pharmacies were not required to provide different interventions from their usual practice, they could / could not be active in this program.

Control pharmacies were eligible to participate in the trial if they provided written authorization, thereby accepting to recruit patients as per the invitation and study leaflet, provide informed consent as per the study leaflet, and follow confidentiality, privacy regulations, and ethics.
