# Supplementary material for: Real-World Effectiveness in Hypertension and Hyperlipidemia Collaborative Management between Pharmacies and Primary Care in Portugal: A Multicenter Pragmatic Controlled Trial (USFarmácia®)
Source: Int J Environ Res Public Health. 2023 Aug 1;20(15):6496. doi: 10.3390/ijerph20156496 (PMC10418740; doi:10.3390/ijerph20156496)
Supplement: Supplementary file 1 [file ijerph-20-06496-s001.zip › ijerph-2232987-supplementary - for XML/SUPPLEMENTARY FILE 2.pdf]

USFarmácia

Juntos cuidamos melhor

HIPERTENSÃO ARTERIAL E/OU DISLIPIDEMIA RISCO CARDIOVASCULAR

- É utente da USF S. Julião?
- Tem idade igual ou superior a 18 anos?
- Toma algum medicamento para a tensão arterial e/ou para o colesterol?
- Ou se não toma nenhum daqueles medicamentos, tem diabetes ou algum outro factor de risco cardiovascular?

SE RESPONDEU SIM, ENTÃO ESTE PROJETO É PARA SI.

A Unidade de Saúde Familiar (USF) S. Julião está a promover um Projecto-Piloto de colaboração com esta Farmácia, baseado numa melhor comunicação entre farmacêuticos, médicos e enfermeiros com a finalidade de melhorar o estado de saúde dos **utentes adultos a tomar medicamentos para a hipertensão arterial e/ou colesterol ou sem este(s) medicamento(s) mas com factores de risco cardiovascular.** O Projecto contempla uma vertente de avaliação, sendo também, por isso, um Estudo de Investigação, a realizar pelo Centro de Estudos e Avaliação em Saúde (CEFAR) da Associação Nacional das Farmácias.

Este Estudo foi notificado à Comissão Nacional de Protecção dos Dados (CNPD) e recebeu pareceres favoráveis da Comissão de Ética da Administração Regional de Saúde (ARS) Centro e do Instituto de Bioética da Universidade Católica Portuguesa (Porto).

SE DER AUTORIZAÇÃO PARA PARTICIPAR NESTE ESTUDO, IRÁ PARTICIPAR NO PROJECTO-PILOTO DE COLABORAÇÃO ENTRE O CENTRO DE SAÚDE E ESTA FARMÁCIA E ESTA FARMÁCIA, O QUE LHE PERMITIRÁ:

- Avaliar os seus valores de tensão arterial, colesterol (total, HDL e LDL) e triglicerídeos.
- Avaliar o seu risco cardiovascular global.
- Beneficiar de um contacto privilegiado entre o seu Médico e Farmacêutico por forma a agendar consulta médica na USF de acordo com o que está definido para o projecto e sempre que necessário.

Se der autorização para participar neste Estudo, irá ser contactado(a) por telefone por um investigador do Centro de Estudos e Avaliação em Saúde (CEFAR) logo no início do Estudo, ao fim de 3, 6 e 12 meses, para responder a umas perguntas sobre os seus factores de risco cardiovascular incluindo tensão arterial e/ou colesterol (total, HDL e LDL) e triglicerídeos, os seus eventuais medicamentos para a tensão alta e/ou colesterol, a sua utilização de cuidados de saúde (ex: consultas, urgências, internamentos, etc) e outras questões relacionadas.

Irá também permitir que o seu Médico da Unidade de Saúde Familiar e o seu Farmacêutico enviem ao CEFAR os seus dados de saúde e dados de eventuais medicamentos relacionados com a hipertensão arterial ou colesterol adquiridos nesta Farmácia.

A SUA PARTICIPAÇÃO É FUNDAMENTAL

A sua participação é fundamental e pode ajudar a conhecer os resultados em saúde dos medicamentos e outras intervenções dos profissionais de saúde, com o intuito de ajudar os médicos, enfermeiros e farmacêuticos a melhorar a resposta às suas necessidades em saúde, em especial à saúde do seu coração.

MUITO IMPORTANTE

- De acordo com a lei Portuguesa, os seus dados serão mantidos confidenciais e nunca será pessoalmente identificado(a) no relatório ou na publicação do Estudo.
- A sua participação é voluntária e só responde às perguntas que quiser.
- Mesmo que aceite participar, em qualquer momento pode recusar a sua participação no Estudo, sem que isso prejudique os serviços que recebe na Farmácia ou na Unidade de Saúde Familiar.

Para dar o seu consentimento, terá que ler, preencher e assinar a **Declaração de Consentimento Informado**, entregando-a ao seu Farmacêutico.

Se tiver alguma questão, pode entrar em contacto com:

Sónia Romano – Farmacêutica e Investigadora Sénior do Estudo
CEFAR - Centro de Estudos e Avaliação em Saúde
Rua Marechal Saldanha, 1 – 1249-069 Lisboa
Tel.: 213 400 670 / E-mail: cefar@anf.pt

A SUA PARTICIPAÇÃO NESTE PROJECTO É IMPORTANTE.
MUITO OBRIGADO PELA COLABORAÇÃO!
