# Supplementary material for: Real-World Effectiveness in Hypertension and Hyperlipidemia Collaborative Management between Pharmacies and Primary Care in Portugal: A Multicenter Pragmatic Controlled Trial (USFarmácia®)
Source: Int J Environ Res Public Health. 2023 Aug 1;20(15):6496. doi: 10.3390/ijerph20156496 (PMC10418740; doi:10.3390/ijerph20156496)
Supplement: Supplementary file 1 [file ijerph-20-06496-s001.zip › ijerph-2232987-supplementary - for XML/SUPPLEMENTARY FILE 3.pdf]

SUPPLEMENTARY FILE 3. List of study sites

1. Intervention sites

Intervention site	Municipality (District)
1. USF S. Julião da Figueira (USF)	Figueira da Foz (Coimbra)
2. Farmácia dos Condados (Pharmacy)	Figueira da Foz (Coimbra)
3. Farmácia Faria (Pharmacy)	Figueira da Foz (Coimbra)
4. Farmácia Garcia (Pharmacy)	Figueira da Foz (Coimbra)
5. Farmácia Gaspar (Pharmacy)	Figueira da Foz (Coimbra)
6. Farmácia Goes Pinheiro (Pharmacy)	Figueira da Foz (Coimbra)
7. Farmácia Jardim (Pharmacy)	Figueira da Foz (Coimbra)
8. Farmácia Saúde (Pharmacy)	Figueira da Foz (Coimbra)

2. Control geographical locations

5 Control USFs and 13 Control Pharmacies located in the following districts: Guarda, Santarém and Viseu.

Glossary:

USF (‘Unidade de Saúde Familiar’): Primary Care Unit (PCU).
