# Supplementary material for: Real-World Effectiveness in Hypertension and Hyperlipidemia Collaborative Management between Pharmacies and Primary Care in Portugal: A Multicenter Pragmatic Controlled Trial (USFarmácia®)
Source: Int J Environ Res Public Health. 2023 Aug 1;20(15):6496. doi: 10.3390/ijerph20156496 (PMC10418740; doi:10.3390/ijerph20156496)
Supplement: Supplementary file 1 [file ijerph-20-06496-s001.zip › ijerph-2232987-supplementary - for XML/SUPPLEMENTARY FILE 4.pdf]

SUPPLEMENTARY FILE 4. Description of Intervention Package (as planned) based on the TIDieR (Template for Intervention Description and Replication) Checklist

Brief Name	Why	What	Who Provided	How	Where	When and How Much	Tailoring	Modifications	How Well
**POC**	Defined in ICP to monitor the effectiveness	BP, TC, lipid profile as per training	Pharmacists, GPs and nurses	Face-to-face	Pharmacy and PCU	Baseline prior to recruitment + follow-up per ICP at the pharmacy, at the discretion of GPs and nurses at PCU	Tailored as per ICP (IT decision algorithm, some flexibility in time intervals	As described in manuscript (Results, Protocol deviations)	Strategies used to improve fidelity in manuscript (Methods). Fidelity data collected for future study
**CVR assessment**	Defined in ICP as hypertension and hyperlipidemia are risk factors of CVR	CVR using SCORE as per training	Pharmacists, GPs and nurses	Face-to-face	Pharmacy and PCU	Per ICP at the pharmacy, at the discretion of GPs and nurses at PCU	Tailored as per ICP (IT decision algorithm), some flexibility in time intervals	As described in manuscript (Results, Protocol deviations)	Strategies used to improve fidelity in manuscript (Methods). Fidelity data collected for future study
**Medication management**	Defined in ICP to enable medication adjustments by GP	Checking for adherence, effectiveness, safety issues focus on anti-hypertensive and lipid-lowering therapy	Pharmacists, GPs and nurses	Face-to-face	Pharmacy and PCU	At every pharmacy appointment and when dispensing repeat medication, at the discretion of GPs at PCU	No	No	Strategies used to improve fidelity in manuscript (Methods). Fidelity data collected for future study
**Referral and request for medical appointment**	Defined in ICP to enable medication adjustments by GP	Request for medical appointment as per ICP	Pharmacists	IT pharmacy to PCU	Pharmacy	Per ICP	Tailored as per ICP (IT decision algorithm), some flexibility	As described in manuscript (Results, Protocol deviations)	Strategies used to improve fidelity in manuscript (Methods). Fidelity data collected for future study

							in time intervals		
**Lifestyle counselling**	Defined in ICP as complementary intervention	Counseling on lifestyle modification as per ICP	Pharmacists, GPs and nurses	Face-to-face	Pharmacy and PCU	At every medical and pharmacy appointment and when dispensing repeat medication	Tailored as per ICP (IT decision algorithm)	No	Strategies used to improve fidelity in manuscript (Methods). Fidelity data collected for future study.
**Request for repeat prescription**	Defined in ICP to minimize adherence gaps	Requesting repeat prescription on behalf of patient	Pharmacists	IT pharmacy to PCU	Pharmacy	When dispensing the last package of anti-hypertensive or lipid-lowering medicine	No	As described in manuscript (Results, Protocol deviations)	No
**Feedback and follow-up**	Defined in ICP to enable feedback from GP and follow-up by pharmacist	Providing feedback to pharmacist	GPs	IT PCU to pharmacy	PCU	After medical appointment requested by pharmacist	At the discretion of GP	As described in manuscript (Results, Protocol deviations)	Strategies used to improve fidelity in manuscript (Methods). Fidelity data collected for future study.
**Refill text reminder to patient**	Defined in ICP to maximize adherence	Sending SMS to patient before end of last package	Pharmacists	IT pharmacy to patient smartphone	Pharmacy	After dispensing repeat medication + 10 days before end of last dispensed package	No	No	No
**Quality Circles (QC)**	Defined in ICP to foster interprofessional collaboration and trust	Interprofessional meetings	Pharmacists, GPs, nurses, trial monitoring team, trial research team.	Face-to-face	Hotel near PCU & pharmacies	Kick-off QC + regular QC, as needed	Flexible approach, as needed	As described in manuscript (Results, Protocol deviations)	No
**Reporting ADEs**	Requested by research team, as per legislation	Formal notification in case of a suspected ADE	Pharmacists - research team - INFARMED	Telephone trial monitoring, email to research team, report in INFARMED website	Pharmacy, research team HQ	Notification to INFARMED within 48 hrs.	No	No	No

POC: Point-of-care measurements; QC: Quality Circles; ADE: Adverse Drug Event; ICP: Integrated Care Pathway (embedded in pharmacy software); CVR: cardiovascular Risk Assessment; GP: General Practitioner; BP: Blood Pressure; TC: Total Cholesterol; IT: Information Technology; PCU: Family Health Unit (primary care unit); HQ: Headquarters.

Example of innovative intervention components in pharmacy software as part of Integrated Care Pathway (ICP)

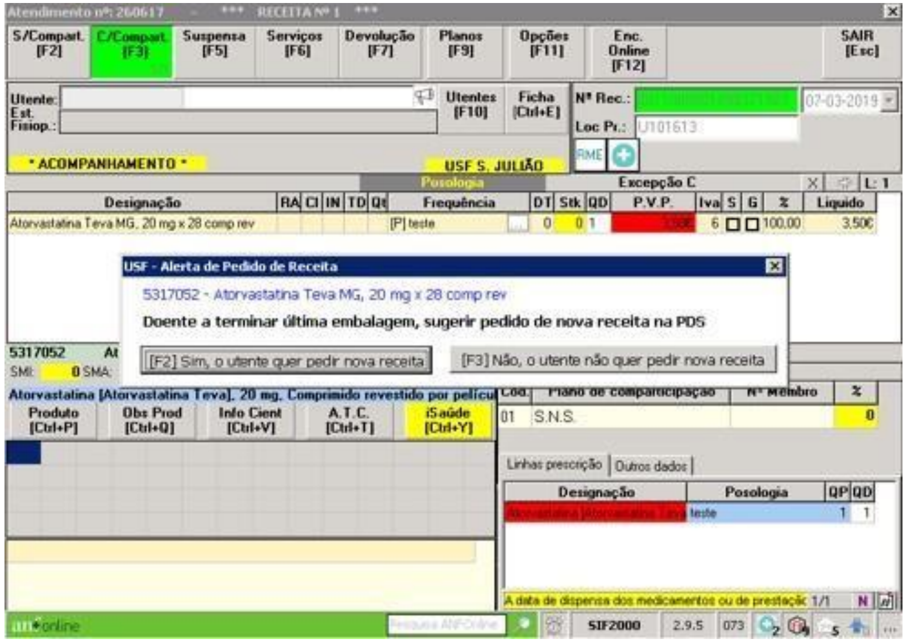

S4FIGURE 1 | Reminder in pharmacy software to request a repeat prescription to PCU

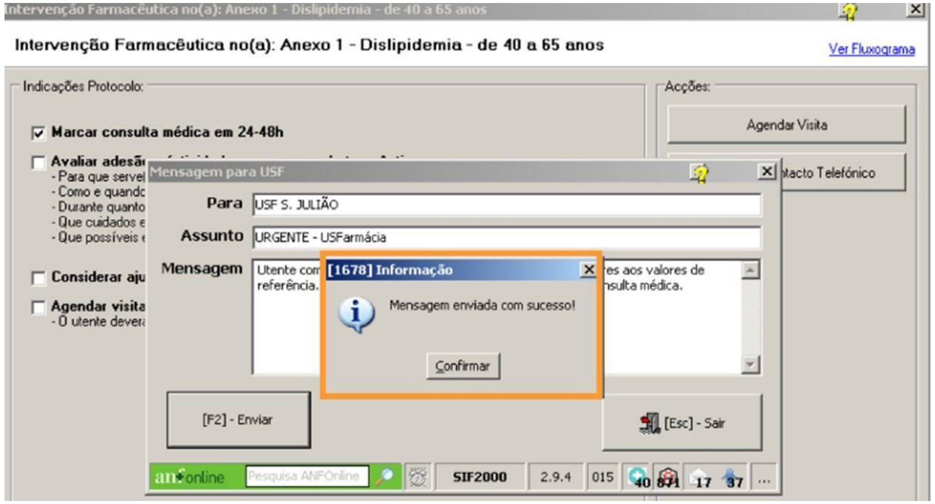

S4FIGURE 2 | Request for a medical appointment sent from pharmacy software to PCU
