# Supplementary material for: Real-World Effectiveness in Hypertension and Hyperlipidemia Collaborative Management between Pharmacies and Primary Care in Portugal: A Multicenter Pragmatic Controlled Trial (USFarmácia®)
Source: Int J Environ Res Public Health. 2023 Aug 1;20(15):6496. doi: 10.3390/ijerph20156496 (PMC10418740; doi:10.3390/ijerph20156496)
Supplement: Supplementary file 1 [file ijerph-20-06496-s001.zip › ijerph-2232987-supplementary - for XML/SUPPLEMENTARY FILE 5.pdf]

TRANSLATED VERSION (Original in Portuguese)

USFarmáciaWatch Monitoring Feedback Report 12

PHARMACY AAA 00000

RECRUITED PATIENTS (Until 29-07-2019)

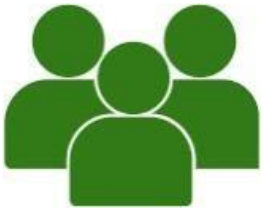

76

PATIENTS RECRUITED IN MY PHARMACY

64

PATIENTS RECRUITED FOR THE STUDY (HYPERLIPIDEMIA, HYPERTENSION, CVR)

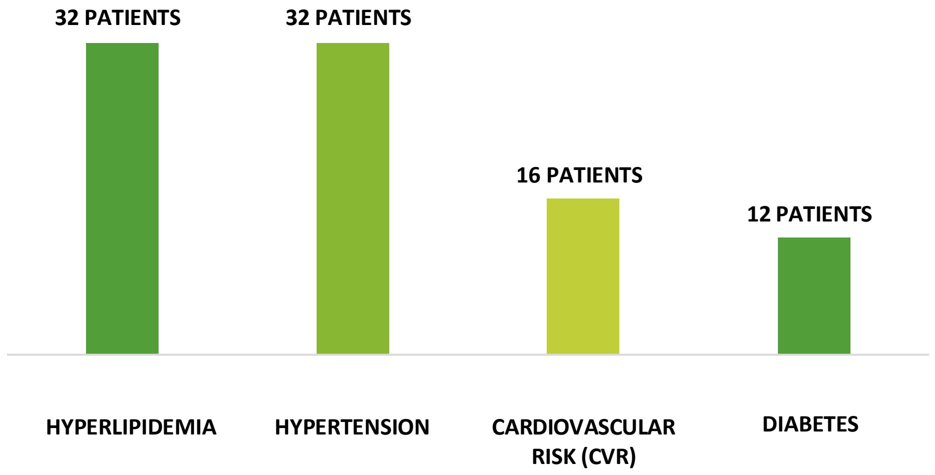

% OF UNCONTROLLED PATIENTS BASELINE VS LAST MEASUREMENT IN MY PHARMACY

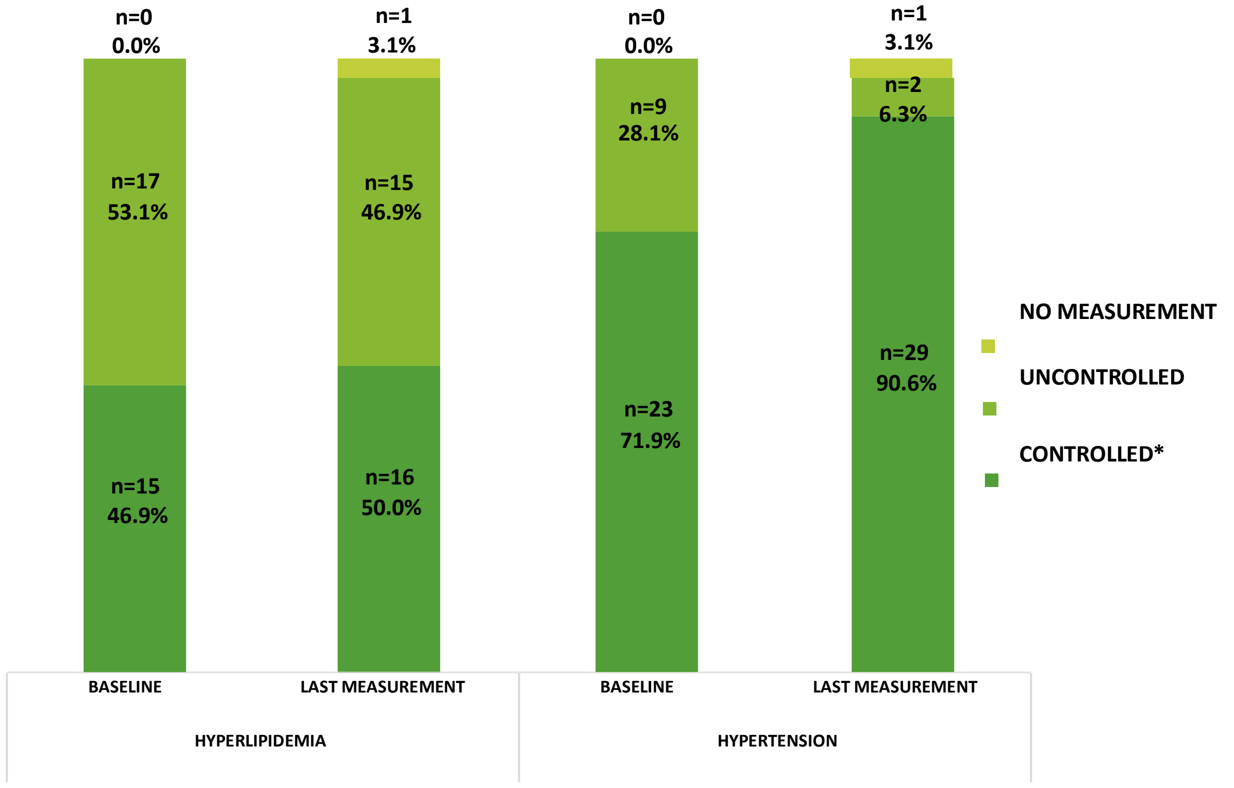

*As in last measurement

USFarmáciaWatch Monitoring
Feedback Report 12

PHARMACY AAA **00000**

FOLLOW-UP OF STUDY PATIENTS

PATIENTS (INITALS+ NHS NO. TRUNCATED)	STUDY CONDITIONS	DATE OF RECRUITMENT	HYPERLIPIDEMIA			HYPERTENSION			DATE OF MEDICAL APPOINTMENT REQUESTED	STATUS OF MEDICAL APPOINTMENT	NEXT SCHEDULED APPOINTMENT IN PHARMACY	STATUS OF APPOINTMENT IN PHARMACY
			NO. MEASUREMENTS	C-LDL*	TC*	NO. MEASUREMENTS	SBP*	DBP*				
GS288XXXX04	CARDIOVASCULAR RISK	16-05-2018	2	-	296	1	117	73	16-03-2019		12-09-2019	12-09-2019
AG275XXXX95	CARDIOVASCULAR RISK	17-05-2018	3	58	192	2	112	74			27-05-2019	Reschedule
FA287XXXX79	HYPERLIPIDEMIA	17-05-2018	2	85	175	2	132	78			07-05-2019	Reschedule
JS271XXXX88	HYPERLIPIDEMIA HYPERTENSION	18-05-2018	2	91	147	5	139	80	25-05-2018	Performed	12-06-2019	Reschedule
JC273XXXX24	HYPERLIPIDEMIA	21-05-2018	3	103	190	3	130	78	22-11-2018		27-05-2019	Reschedule
CL281XXXX32	HYPERLIPIDEMIA HYPERTENSION	22-05-2018	2	118	191	3	120	80	20-12-2018	Performed	18-06-2019	Reschedule
AA277XXXX42	HYPERLIPIDEMIA	24-05-2018	3	90	181	2	105	72			24-05-2019	Reschedule
AP292XXXX03	HYPERTENSION	25-05-2018	-	-	-	4	117	70			29-04-2019	Reschedule
MT279XXXX86	HYPERLIPIDEMIA	30-05-2018	2	85	209	1	125	86			30-11-2019	30-11-2019
NT279XXXX27	CARDIOVASCULAR RISK	30-05-2018	1	78	157	3	122	65			12-06-2020	12-06-2020
ML281XXXX96	HYPERLIPIDEMIA HYPERTENSION	31-05-2018	2	123	206	3	102	65			01-06-2019	Reschedule
SS281XXXX88	HYPERLIPIDEMIA HYPERTENSION	31-05-2018	2	77	136	2	91	48			01-06-2019	Reschedule
JD363XXXX47	HYPERTENSION	01-06-2018	-	-	-	3	100	78			06-09-2019	06-09-2019
OD367XXXX84	CARDIOVASCULAR RISK	01-06-2018	1	-	182	3	113	95	07-06-2018	Performed	27-05-2020	27-05-2020
AG282XXXX74	HYPERLIPIDEMIA HYPERTENSION	04-06-2018	3	-	218	3	123	72			04-06-2019	Reschedule
RP273XXXX97	HYPERTENSION	04-06-2018	-	-	-	3	120	87			19-06-2019	Reschedule
AM278XXXX50	HYPERTENSION	06-06-2018	1	-	161	5	130	74			11-01-2020	11-01-2020
OP287XXXX38	HYPERTENSION	07-06-2018	-	-	-	3	110	73			18-06-2019	Reschedule
JD288XXXX78	HYPERLIPIDEMIA	08-06-2018	2	112	167	1	99	69			29-06-2019	Reschedule
AC281XXXX54	HYPERLIPIDEMIA HYPERTENSION	12-06-2018	5	82	158	3	139	82	21-12-2018	Performed	19-06-2019	Reschedule
SS293XXXX40	HYPERLIPIDEMIA	13-06-2018	2	137	214	1	104	82	21-12-2018		19-06-2019	Reschedule
MS282XXXX90	HYPERLIPIDEMIA HYPERTENSION	14-06-2018	1	89	185	1	111	76			15-06-2019	Reschedule
DC285XXXX56	CARDIOVASCULAR RISK	16-06-2018	6	-	218	5	139	80			09-07-2020	09-07-2020
PS290XXXX36	HYPERTENSION	21-06-2018	-	-	-	5	162	87	28-05-2019		19-01-2020	19-01-2020
HV289XXXX76	HYPERLIPIDEMIA HYPERTENSION	03-07-2018	3	56	136	3	119	85			25-06-2019	Reschedule
MC273XXXX08	HYPERLIPIDEMIA	05-07-2018	4	53	152	1	101	63	05-07-2018	Performed	29-01-2019	Reschedule
MA281XXXX70	HYPERLIPIDEMIA HYPERTENSION	13-07-2018	2	-	173	2	138	71			30-07-2019	Schedule appointment
AS210XXXX30	HYPERTENSION	16-07-2018	-	-	-	2	113	74			24-09-2019	24-09-2019
MM278XXXX05	HYPERLIPIDEMIA HYPERTENSION	16-07-2018	3	106	191	3	120	72			11-01-2020	11-01-2020

*Last measurement

cefar
centro de estudos e avaliação em saúde

PHARMACY AAA **00000**

FOLLOW-UP OF STUDY PATIENTS

PATIENTS (INITALS+ NHS NO. TRUNCATED)	STUDY CONDITIONS	DATE OF RECRUITMENT	HYPERLIPIDEMIA			HYPERTENSION			DATE OF MEDICAL APPOINTMENT REQUESTED	STATUS OF MEDICAL APPOINTMENT	NEXT SCHEDULED APPOINTMENT IN PHARMACY	STATUS OF APPOINTMENT IN PHARMACY
			NO. MEASUREMENTS	C-LDL*	TC*	NO. MEASUREMENTS	SBP*	DBP*				
RF279XXXX03	HYPERLIPIDEMIA HYPERTENSION	16-07-2018	2	94	184	2	106	75			03-09-2019	03-09-2019
DF280XXXX95	HYPERLIPIDEMIA	17-07-2018	2	-	240	1	118	79			01-08-2019	01-08-2019
FP287XXXX61	HYPERTENSION	18-07-2018	-	-	-	2	122	84			23-09-2019	23-09-2019
MB282XXXX04	HYPERLIPIDEMIA HYPERTENSION	19-07-2018	2	124	197	2	109	72			23-07-2019	Reschedule
NP281XXXX10	CARDIOVASCULAR RISK	19-07-2018	2	148	251	1	127	81			01-10-2019	01-10-2019
AL281XXXX26	HYPERLIPIDEMIA	20-07-2018	3	121	168	3	88	55			23-07-2019	Reschedule
MF281XXXX42	HYPERLIPIDEMIA	23-07-2018	2	113	193	2	104	83	02-05-2019		29-10-2019	29-10-2019
ML288XXXX88	HYPERLIPIDEMIA	24-07-2018	2	102	178	2	130	90			31-07-2019	31-07-2019
CR289XXXX85	HYPERTENSION	25-07-2018	-	-	-	2	126	77			22-08-2019	22-08-2019
JR273XXXX16	HYPERLIPIDEMIA HYPERTENSION	25-07-2018	4	182	255	2	128	79	30-01-2019	Performed	29-07-2019	Reschedule
FM291XXXX70	HYPERLIPIDEMIA	26-07-2018	2	-	161	1	115	83			26-07-2019	Reschedule
MP279XXXX25	HYPERLIPIDEMIA HYPERTENSION	27-07-2018	3	-	230	3	136	72	30-01-2019	Performed	18-07-2019	Reschedule
MS281XXXX17	HYPERTENSION	27-07-2018	2	148	220	6	160	73	16-08-2018	Performed	19-11-2019	19-11-2019
MF275XXXX94	HYPERLIPIDEMIA	08-08-2018	2	78	141	2	88	65			08-08-2019	08-08-2019
JB286XXXX79	HYPERTENSION	17-08-2018	-	-	-	2	128	74			30-11-2019	30-11-2019
SB286XXXX87	CARDIOVASCULAR RISK	17-08-2018	1	87	156	1	100	73			16-08-2020	16-08-2020
ES274XXXX61	CARDIOVASCULAR RISK	06-09-2018	1	144	222	1	116	88			06-09-2019	06-09-2019
M282XXXX38	HYPERTENSION	17-09-2018	2	85	186	2	106	67			09-01-2020	09-01-2020
CP276XXXX76	HYPERLIPIDEMIA HYPERTENSION	19-09-2018	4	50	136	5	127	82			08-11-2019	08-11-2019
AC289XXXX49	CARDIOVASCULAR RISK	20-09-2018	2	119	202	1	110	75			02-10-2019	02-10-2019
MP276XXXX68	HYPERLIPIDEMIA	20-09-2018	2	-	203	1	121	66			20-12-2019	20-12-2019
NS284XXXX68	CARDIOVASCULAR RISK	20-09-2018	2	114	195	1	127	90	04-10-2018	Performed	04-11-2019	04-11-2019
AR279XXXX72	HYPERTENSION	24-09-2018	1	101	147	2	114	77			08-11-2019	08-11-2019
TO288XXXX83	CARDIOVASCULAR RISK	27-09-2018	2	83	158	1	135	102	04-10-2018	Performed	05-11-2019	05-11-2019
MN289XXXX17	CARDIOVASCULAR RISK	01-10-2018	1	196	260	1	109	86			31-05-2019	Reschedule
JD281XXXX38	HYPERTENSION	04-10-2018	1	154	244	3	131	83	11-10-2018	Performed	11-10-2018	Reschedule
ET291XXXX59	HYPERTENSION	10-10-2018	1	77	164	2	134	72			12-01-2020	12-01-2020
MR293XXXX77	HYPERTENSION	12-10-2018	1	68	131	4	113	80			12-01-2020	12-01-2020
PL384XXXX77	CARDIOVASCULAR RISK	22-10-2018	2	161	259	1	109	67			13-05-2019	Reschedule

*Last measurement

cefar
centro de estudos e avaliação em saúde

PHARMACY AAA **00000**

FOLLOW-UP OF STUDY PATIENTS

PATIENTS (INITALS+ NHS NO. TRUNCATED)	STUDY CONDITIONS	DATE OF RECRUITMENT	HYPERLIPIDEMIA			HYPERTENSION			DATE OF MEDICAL APPOINTMENT REQUESTED	STATUS OF MEDICAL APPOINTMENT	NEXT SCHEDULED APPOINTMENT IN PHARMACY	STATUS OF NEXT APPOINTMENT IN PHARMACY
			NO. MEASUREMENTS	C-LDL*	TC*	NO. MEASUREMENTS	SBP*	DBP*				
MC278XXXX06	HYPERLIPIDEMIA	24-10-2018	2	-	139	1	131	80			24-10-2019	24-10-2019
MF274XXXX06	HYPERLIPIDEMIA	26-10-2018	2	-	243	1	93	66			26-10-2019	26-10-2019
AJ287XXXX48	CARDIOVASCULAR RISK	31-10-2018	1	-	233	2	131	75			09-11-2019	09-11-2019
MM282XXXX70	HYPERLIPIDEMIA HYPERTENSION	31-10-2018	2	74	158	2	112	82			05-05-2020	05-05-2020
GS282XXXX26	CARDIOVASCULAR RISK	12-11-2018	2	197	285	1	99	68	13-07-2019		09-01-2020	09-01-2020
MR285XXXX78	CARDIOVASCULAR RISK	21-11-2018	1	111	174	1	97	73			21-11-2020	21-11-2020

*Last measurement
