# Supplementary material for: Real-World Effectiveness in Hypertension and Hyperlipidemia Collaborative Management between Pharmacies and Primary Care in Portugal: A Multicenter Pragmatic Controlled Trial (USFarmácia®)
Source: Int J Environ Res Public Health. 2023 Aug 1;20(15):6496. doi: 10.3390/ijerph20156496 (PMC10418740; doi:10.3390/ijerph20156496)
Supplement: Supplementary file 1 [file ijerph-20-06496-s001.zip › ijerph-2232987-supplementary - for XML/SUPPLEMENTARY FILE 6.pdf]

SUPPLEMENTARY FILE 6. Experimental Bundled Payment Model for USFarmácia® Pharmacy Services

Reimbursement Rules and Billing Procedure for USFarmácia® Pharmacy Services

RULES

Program Saúda® will reimburse 100% of the following pharmacy point-of-care assessments and visits of eligible patients in the USFarmácia® Trial according to the following rules:

Code (CNP)	Service	Reimbursement Price
7942623	Blood Pressure Measurement	1.50 €
7953539	Lipid Profile Measurement	14.00 €
7969055	Cardiovascular Risk Assessment	6.25 €
7969162	USFarmácia® Visit_T0	19.56 €
7969170	USFarmácia® Visit_T6	9.78 €
7969188	USFarmácia® Visit_T12	9.78 €
7969196	USFarmácia® Visit_TX	6.52 €

CARDIOVASCULAR RISK

Eligible Patients: Patients 40-65 years with no medication for hypertension or hyperlipidemia and with at least one cardiovascular risk factor, as per Integrated Care Pathway (ICP), and no diabetes or medication for diabetes.

Eligible services per patient:

1 Cardiovascular Risk Assessment 6.25

1 USFarmácia® Visit_T0 (First Visit) 19.56

25.81

Payment cap: 1 pharmacy visit per patient; max. 390 total trial-eligible patients.

HYPERTENSION

Eligible Patients: Patients with no diabetes with at least one medication for hypertension (and baseline blood pressure ≥140/90 mmHg).

Eligible services per patient:

First Visit (T0):

1 Blood Pressure Measurement 1.50

1 USFarmácia® Visit_T0 19.56

21.06

Visit 6 ± 2 Months (T6):

1 Blood Pressure Measurement 1.50

1 USFarmácia® Visit_T6 9.78

11.28

Visit 12 ± 2 Months (T12):

1 Blood Pressure Measurement 1.50

1 USFarmácia® Visit_T12 9.78

11.28

Extra Visit (if required for Blood Pressure Measurement, as per ICP) X± 1 Month (TX):

1 Blood Pressure Measurement 1.50

1 USFarmácia® Visit_TX 6.52

8.02

Payment cap: 3-4 pharmacy visits per patient; max. 208 total trial-eligible patients.

HYPERLIPIDEMIA

Eligible Patients: Patients with no diabetes with at least one medication for hyperlipidemia (and baseline total cholesterol ≥190 mg/dL or c-LDL ≥115 mg/dL).

Eligible services per patient:

First Visit (T0):

1 Lipid Profile Measurement 14.00

1 USFarmácia® Visit_T0 19.56

33.56

Visit 6 ± 2 Months (T6):

1 Lipid Profile Measurement 14.00

1 USFarmácia® Visit_T6 9.78

23.78

Visit 12 ± 2 Months (T12):

1 Lipid Profile Measurement 14.00

1 USFarmácia® Visit_T12 9.78

23.78

Payment cap: 3 pharmacy visits per patient; max. 234 total trial-eligible patients.

OBS: If a patient is simultaneously on hypertension AND hyperlipidemia medication, both point-of-care tests (blood pressure and lipid profile) are eligible for visits T0, T6, and T12.

BILLING PROCEDURE

1. After scheduling the next visit as per ICP: go to menu **SERVICES [F6], identify the eligible patient** and insert **applicable CNP code(s).**
2. Confirm reimbursement price of point-of-care measurements (please change to your usual retail price for your other patients outside this project).

   OBS: Project Pharmacies will receive a plastic card with a CNP code list to affix at each sales counter for a quick reference guide.
3. Go to menu **PLANS [F9]**, select **Saúda**® **Reimbursement Plan (code CS),** and insert the **patient NHS number**.

   OBS: This Plan will not be available for invoicing in usual pharmacy invoice batches.
4. Print invoice/receipt with the total amount (100% reimbursed).
5. Hand invoice/receipt to the patient (patient pays 0,00€).

REIMBURSEMENT TO PHARMACIES

The amount due under this project will appear in the Saúda® invoice to the pharmacy, under "Extras", named "USFarmácia® – Intervenções". Payment to pharmacies will be made using e-reimbursement on two-time points: 1) Saúda® invoice February 2019 comprising project eligible services provided in 2019; 2) Saúda® final invoice at end of study follow-up *(projected date: August 2019).*

Amount billed will be subject to prior validation by Study Team to conform with Rules for Reimbursement of USFarmácia services.

WHICH DOCUMENTS MUST PHARMACIES SEND FOR BILLING AND REIMBURSEMENT?

No document is required – billing and reimbursement will be processed electronically.

WHAT IS THE AMOUNT DUE PER PHARMACY?

The amount due depends on the number of eligible patients who meet inclusion criteria and rules for reimbursement approved for this project.

MORE INFORMATION:

Study Team
Tel. XXXXX / email: XXXX
